# The Mechanisms and Boundary Conditions of Drug Memory Reconsolidation

**DOI:** 10.3389/fnins.2021.717956

**Published:** 2021-08-06

**Authors:** Liangpei Chen, He Yan, Yufang Wang, Ziping He, Qihao Leng, Shihao Huang, Feilong Wu, Xiangyang Feng, Jie Yan

**Affiliations:** ^1^Department of Forensic Science, School of Basic Medical Science, Central South University, Changsha, China; ^2^Xiangya School of Medicine, Central South University, Changsha, China; ^3^Key Laboratory of Molecular Epidemiology of Hunan Province, School of Medicine, Hunan Normal University, Changsha, China; ^4^Department of Forensic Science, School of Basic Medical Science, Xinjiang Medical University, Urumqi, China

**Keywords:** drug memory, addiction, reconsolidation, limbic–corticostriatal system, boundary condition

## Abstract

Drug addiction can be seen as a disorder of maladaptive learning characterized by relapse. Therefore, disrupting drug-related memories could be an approach to improving therapies for addiction. Pioneering studies over the last two decades have revealed that consolidated memories are not static, but can be reconsolidated after retrieval, thereby providing candidate pathways for the treatment of addiction. The limbic–corticostriatal system is known to play a vital role in encoding the drug memory engram. Specific structures within this system contribute differently to the process of memory reconsolidation, making it a potential target for preventing relapse. In addition, as molecular processes are also active during memory reconsolidation, amnestic agents can be used to attenuate drug memory. In this review, we focus primarily on the brain structures involved in storing the drug memory engram, as well as the molecular processes involved in drug memory reconsolidation. Notably, we describe reports regarding boundary conditions constraining the therapeutic potential of memory reconsolidation. Furthermore, we discuss the principles that could be employed to modify stored memories. Finally, we emphasize the challenge of reconsolidation-based strategies, but end with an optimistic view on the development of reconsolidation theory for drug relapse prevention.

## Introduction

**FIGURE 1:**
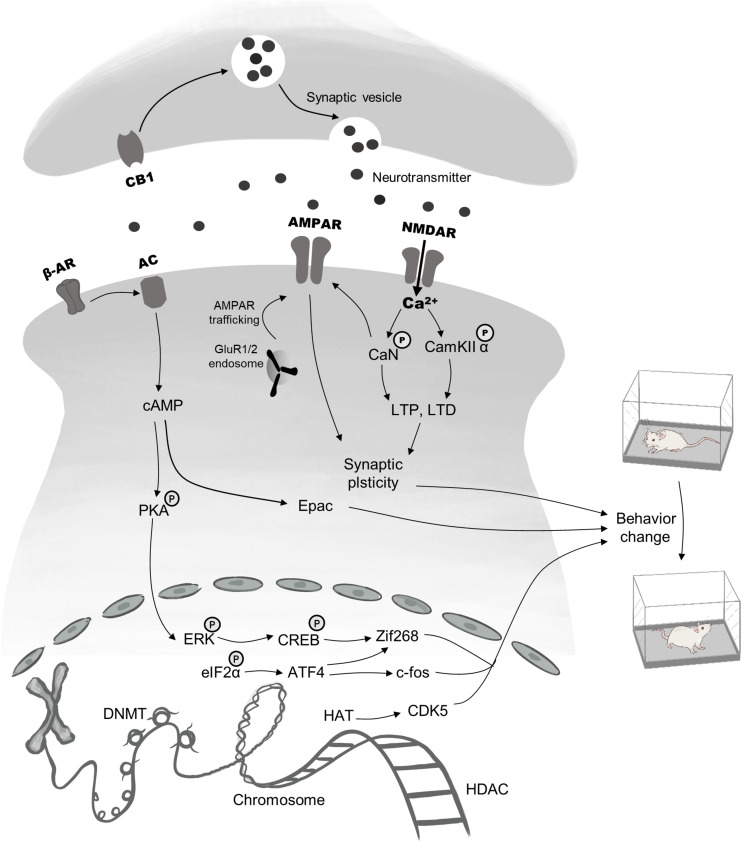
Brief description of pharmacological targets and signaling cascades recruited in reconsolidation. The process of drug memory reconsolidation requires a complicated regulatory network, including epigenetic mechanisms, gene transcription, and activation of membrane receptors, all of which are responsible for behavior changes. Targets for epigenetic modifications mainly lie in the HAT, the HDAC, the DNMT. Besides, the phosphorylation and dephosphorylation of ERK and eIF2α within the nucleus regulate the expression of immediate early genes, such as CREB, Zif 268, and c-fos, thus ultimately lead to changes in addiction behaviors. Finally, pre-and postsynaptic membrane receptors including AMPAR, NMDAR, β-AR, and CB1 have been proved to be effective targets. Main downstream mechanisms contain the second messenger (cAMP)-mediated pathway, AMPAR & NMDAR regulated synaptic plasticity and neurotransmitter transport.

Memory in drug addiction is usually abnormal and is considered to reflect a learning disorder (Phelps and Hofmann, 2019). The central goal of addiction treatments is to prevent relapse and compulsive drug-seeking behavior. Drug-associated memories, therefore, provide an effective treatment target to reduce relapse (Lee et al., 2017). Generally, drug memory can be viewed as a kind of associative memory that combines a conditioned stimulus (CS) with a rewarding drug stimulus [the unconditioned stimulus (US)] (Xue et al., 2012). Researchers have made substantial progress in reducing negative effects related to drugs by disrupting associative memory. One of the factors contributing to this advance is the significant development of the theory of memory reconsolidation. Although early studies indicated that consolidated memory may be diminished after retrieval (Misanin et al., 1968; Przybyslawski and Sara, 1997), the mechanism underlying reconsolidation was not well understood. In 2000, Nader et al. (2000b) found that previously consolidated memories can be labile after retrieval, and that the synthesis of new proteins is necessary for long-term storage—a process they putatively termed reconsolidation. Initial research into reconsolidation mainly focused on fear memory, but because of the promising clinical therapeutic potential of this theory, later studies expanded the research to investigate drug memories with encouraging success (Lee et al., 2005; Xue et al., 2012). Editing well-established memories provides a means by which detrimental memories driving relapse can be disrupted (Xue et al., 2012, 2017b). Meanwhile, studies using a large variety of amnestic agents have contributed to elucidating neural circuits involved in memory updating. Here, we focus on reviewing the limbic–corticostriatal circuits recruited during reconsolidation, in addition to several molecular processes that may serve as potent targets to disrupt reconsolidation (see Figure 1). In addition, we discuss the boundary conditions that limit reconsolidation-based strategies.

## Structures in the Brain Related to Reconsolidation

Several brain regions interact to form CS–US associative memories, thereby directing reward-seeking behaviors. Limbic-corticostriatal circuitry, including the amygdala, hippocampus, striatum, and prefrontal cortex, are required to form associative memories between stimuli and rewards. The underlying molecular mechanisms contributing to CS–US association memories in these brain areas and circuits are listed in Table 1.

**TABLE 1:** Summary of experiments addressing mechanisms of Pavlovian memory reconsolidation.

**Brain area**	**Behavioral paradigm**	**Species**	**Drug**	**Target**	**Treatment**	**Effect**	**References**
LA	SA	Rat	Cocaine	CaN	CGA	Disruption	Rich et al., 2020
LA	SA	Rat	Cocaine	Histone deacetylase	Inhibit (trichostatn A)	Enhance	Monsey et al., 2019
BLA	SA	Rat	Cocaine	DNA methyltransferase	Inhibit (5-azacytidine)	Disruption	Shi et al., 2015
BLA	CPP, SA	Rat	Morphine Heroin	eIF2α	Sal003	Disruption	Jian et al., 2014
BLA	SA	Rat	Cocaine	PKA	Rp-cAMPS,	Disruption	Arguello et al., 2014
BLA	SA	Rat	Cocaine	Epac	8-CPT	Disruption	Wan et al., 2014
BLA	SA	Rat	Cocaine	CaMKIIα	KN-93 or KN-62	Disruption	Rich et al., 2016
BLA	SA	Rat	Cocaine	Zif268	ASO	Disruption	Lee et al., 2005, 2006
BLA	SA	Rat	Cocaine	ERK	U0126	Disruption	Wells et al., 2013
BLA	CPP	Rat	Cocaine	CDK5	beta-butyrolactone	Disruption	Li et al., 2010
BLA	SA	Rat	Cocaine	NMDAR	D-APV	Disruption	Milton et al., 2008
BLA	CPP	Rat	Cocaine	β-AR	propranolol	Disruption	Otis et al., 2013
BLA	CPP	Rat	Morphine	GRs	GR agonist	Disruption	Wang et al., 2008
BLA	SA	Rat	Cocaine	NMDAR	D-APV	Disruption	Milton et al., 2008
BLA	CPP	Rat	Morphine	β-AR	Propranolol	No effect	Wu et al., 2014
BLA	CPP	Rat	Morphine	Protein synthesis	Anisomycin	No effect	Yim et al., 2006
BLA	SA	Rat	Cocaine	CaMKII	KN-93	No effect	Arguello et al., 2014
CeA	CPP	Mice	Cocaine	β2-AR	ICI 118, 551	No effect	Zhu et al., 2018
CeA	SA	Rat	Alcohol	mTORC1	Rapamycin	Disruption	Barak et al., 2013
CeA	CPP	Rat	Cocaine	CDK5	beta-butyrolactone	No effect	Li et al., 2010
DH	CPP	Mice	Cocaine	DNA demethylation	Knockdown (Tet3)	Disruption	Liu et al., 2018
NAc	SA	Rat	Cocaine	DNA methyltransferase	Inhabit (RG108)	Disruption	Massart et al., 2015
NAc	SA	Rat	Cocaine	DNA methyltransferase	Enhance (S-adenosylmethionine)	Disruption	Massart et al., 2015
NAc core	CPP, SA	Rat	Cocaine	calpain	calpain inhibitor	Disruption	Liang et al., 2017
NAc	SA	Rat	Cocaine	ERK	U0126	No effect	Wells et al., 2013
mPFC	CPP	Rat	Cocaine	PNNs	Ch-ABC	Disruption	Slaker et al., 2015
PL-mPFC	CPP	Rat	Cocaine	β-AR	Propranolol, nadolol	Disruption	Otis et al., 2013
system	CPP	Rat	Morphine	GR	stress	Disruption	Wang et al., 2008
system	SA	Rat	Cocaine	Histone acetyltransferase	Inhibit (garcinol)	Disruption	Dunbar and Taylor, 2017
system	SA	Rat	Cocaine	mTOR	rapamycin	Disruption	Zhang et al., 2021
system	SA	Rat	Cocaine	CB1R	AM251	Disruption	Higginbotham et al., 2021b
system	CPP	Rat	Heroin	β-AR	propranolol	Disruption	Chen et al., 2021

*AM251, CB1R antagonist; anisomycin, protein synthesis inhibitor; ASO, anti-sense oligodeoxynucleotides; beta-butyrolactone, CDK5 inhibitor; β-AR, β-adrenergic receptor; BLA, basolateral amygdala; CaMKII, calcium-calmodulin-dependent kinase II; CaN, calcineurin; CB1R, cannabinoid type 1 receptor; CDK5, neuronal protein kinase cyclin-dependent kinase 5; CeA, central amygdala; CGA, chlorogenic acid; Ch-ABC, chondroitinase-ABC; CPP, conditioned place preference; D-APV, D(−)-2-amino-5-phosphonopentanoic acid (NMDAR antagonist); DH, dorsal hippocampus; 8-CPT, 8-(4-chlorophenylthio)-2′-O-methyladenosine-3′, 5′-cyclic monophosphate; Epac, (Exchange Protein Activated by cAMP)-specific agonist; eIF2α, eukaryotic initiation factor 2α; Epac, exchange protein activated by cAMP; ERK, extracellular signal-regulated kinase; garcinol, histone acetyltransferase inhibitor; GR, glucocorticoid; stress, 5-min forced swim in ice-cold water; ICI-118,551, a β2-AR selective antagonist; KN-93 and KN-62, CaMKII inhibitors; LA, lateral amygdala; mTORC1, mammalian target of rapamycin complex 1; NAc, nucleus accumbens; NMDAR, N-methyl-D-aspartate receptor; PKA, protein kinase A; PNNs, perineuronal nets; propranolol, non-specific β-AR inhibitor; rapamycin, mTORC1 inhibitor; Rp-cAMPS, Rp-adenosine 3′,5′-cyclic monophosphorothioate triethylammonium salt (PKA inhibitor); Sal003, a selective inhibitor of eIF2α dephosphorylation; Tet3, ten-eleven translocation methylcytosine dioxygenase 3; RG108, DNA methyltransferase inhibitor; SA, self-administration; S-adenosylmethionine, methyl donor; U0126, ERK inhibitor; Zif268, immediate-early gene, also known as EGR1, NGFI-A, and Krox24.*

### Amygdala Is Required for Memory Retrieval

The amygdala is a brain structure that plays a critical role in emotion and motivation (Blundell et al., 2001) and that is actively involved in processing rewarding environmental stimuli (Janak and Tye, 2015). In terms of the subregions of the amygdala, the basolateral amygdala (BLA) is a key brain structure involved in CS-induced memory reconsolidation (Higginbotham et al., 2021a,b). BLA neurons store the associative emotional learning engrams that are recruited during retrieval (Pignatelli et al., 2019). BLA neurons receive dopaminergic input from the ventral tegmental area (VTA), and project to the nucleus accumbens (NAc) via glutamatergic neurons, contributing to the process underlying the incubation of craving (Lüscher, 2016). Remarkably, the central nucleus of the amygdala (CeA) and the BLA play different roles in memory reconsolidation (Kruzich and See, 2001). For example, Jian et al. (2014) found that CS-induced reconsolidation of morphine and cocaine memories in rats could be disrupted by selectively inhibiting the dephosphorylation of the eukaryotic initiation factor 2 α-subunit (eIF2α) in the BLA but not in the CeA. However, the CeA may play an essential role in US-induced drug memory reconsolidation. In another study, researchers found that US-induced but not CS-induced cocaine memory reconsolidation required β1-adrenergic signaling and *de novo* protein synthesis in the CeA, indicating that the CeA may be required for US retrieval but not CS retrieval (Zhu et al., 2018).

### Hippocampus Is Required for the Storage of Drug-Paired Context

The hippocampus is known to organize episodic memory and is required for the formation of Pavlovian conditioned associations (also known as classical conditioning), as measured by conditioned place preference (CPP) (Taubenfeld et al., 2010; Liu et al., 2018). In terms of operant drug-seeking behavior, the hippocampus seems to be less directly required, as the cue or tone represents much less of a spatial object (Fuchs et al., 2005). Besides, the hippocampus does not appear to encode the memory trace of the conditioning context alone, as microinjections of the protein synthesis inhibitor anisomycin into the dorsal hippocampus (DH) do not disrupt cocaine memory reconsolidation (Ramirez et al., 2009). In contrast, contralateral BLA microinjections of the protein synthesis inhibitor baclofen/muscimol disrupt cocaine memory reconsolidation, suggesting that interaction between the DH and BLA is involved in editing the context–drug engram (Wells et al., 2011). Moreover, using optogenetic techniques, researchers found that the dorsal CA1 (dCA1) subregion of the hippocampus directly projects to the NAc, indicating that the spatial memory trace facilitates effective appetitive behavior via a limbic–motor interface (Trouche et al., 2019). Taken together, these studies demonstrate that the hippocampus is required for reward-motivated behavior and that it does not mediate reconsolidation alone.

### Striatum Drives Cue–Reward Learning

The striatum is necessary for learning that actions result in reward and for executing actions. In rodents, the striatum is typically divided into three main subregions: dorsolateral striatum (DLS), dorsomedial striatum (DMS), and ventral striatum (VS) (Cox and Witten, 2019). The DLS plays a vital role in stimulus–response association, which is necessary for the formation of skills and for habituation (Barnes et al., 2005; O’Hare et al., 2016). In contrast, the DMS is involved in goal-directed behaviors that depend on response–outcome associations (Yin et al., 2005). In fact, there is a shift of action transitions from goal-directed to habitual after overtraining (Thorn et al., 2010). Both DLS and DMS are recruited in the process of drug-seeking and consumption, which rely on instrumental learning. Early studies proved that instrumental learning does not require protein synthesis-dependent memory reconsolidation (Hernandez and Kelley, 2004; Brown et al., 2008). However, recently it has been revealed that instrumental memories for drug addiction (e.g., to cocaine or nicotine) may go through reconsolidation (Exton-McGuinness et al., 2014, 2019; Piva et al., 2020), providing a new perspective on how to reduce drug abuse behaviors. With regard to the VS, its primary function can be attributed to its major component, the NAc, which is essential for the formation of stimulus–outcome associations in Pavlovian learning (Milton and Everitt, 2012). The NAc receives dopaminergic neuronal input from the VTA, which plays a key role in processing reward stimuli (Nutt et al., 2015). In addition, glutamatergic neurons in the BLA, prefrontal cortex (PFC), and ventral hippocampus also project to the NAc, contributing to its crucial role in drug-evoked synaptic plasticity (Lüscher, 2016).

### Prefrontal Cortex Modulates Reward Circuits

Drug addiction was initially thought to be caused by the dysfunction of subcortical reward circuits. However, accumulating evidence indicates that the PFC is recruited during drug addiction via regulation of limbic reward regions (Goldstein and Volkow, 2011). The PFC is necessary for action selection and decision making based on the value of goals (Hyman et al., 2006; Szczepanski and Knight, 2014). Research has shown that associative learning during cocaine abuse induces plasticity in medial prefrontal cortex (mPFC) neurons to alter the reward system (Porter and Sepulveda-Orengo, 2020). Furthermore, the removal of perineuronal nets, which play an essential role in neural plasticity, from GABAergic interneurons modulating the activity of pyramidal neurons in the PFC impaired the reconsolidation of a cocaine CPP (Slaker et al., 2015), suggesting that the PFC is required for drug memory reconsolidation. In another study, β-adrenergic receptor (β-AR) blockade in the prelimbic medial prefrontal cortex (PL-mPFC) persistently reduced the expression of a cocaine CPP memory when administered before, but not after, cocaine memory retrieval (Otis et al., 2013). This indicates that blockade of the β-ARs disrupted the retrieval, but not the reconsolidation, of cocaine CPP memory. These studies illustrate the sophisticated role played by the PFC in the process of memory reconsolidation.

There are limitations in the conclusions that can be drawn from circuit-based studies targeting specific brain regions using lesion or inactivation, as these methods may disrupt neural communication between structures. The mechanisms underlying reconsolidation remain to be further clarified considering the neuronal projections within the limbic–corticostriatal system. With the advance of neuronal manipulation techniques, such as the newly developed wireless optogenetic technique (Yang et al., 2021), we are optimistic that these limitations can be addressed in future work.

## Potential Targets for Relapse Prevention

While the brain areas required for associating environmental cues with drug stimuli have been well established, the underlying mechanisms of memory destabilization and re-stabilization at the molecular and synaptic levels are poorly understood. Here, we focus on reviewing novel potent targets for modulating drug memory reconsolidation.

### Function of Epigenetic Mechanisms in Preventing Drug Relapse

Accumulating evidence indicates that epigenetic regulation plays a critical role in the process of drug-induced neuronal plasticity (Renthal and Nestler, 2008; Werner et al., 2021), which can involve long-lasting changes in gene expression and ultimately result in behavioral changes (Feng and Nestler, 2013). Here, we focus on the use of DNA demethylation and histone deacetylation during memory destabilization and their impact on relapse prevention.

DNA methylation in the VTA has been shown to play a role in associative memory combining environmental cues with drug reward (Day et al., 2013). Although DNA methylation was initially perceived as a stable process that cannot be rapidly modulated, subsequent studies have demonstrated that this is not the case (Miller et al., 2010; Zipperly et al., 2021). In the brain, DNA undergoes rapid methylation and demethylation, which is necessary for memory formation and synaptic plasticity (Feng et al., 2010; Miller et al., 2010; Day et al., 2013). This provides an avenue for disrupting drug memory during the reconsolidation window. For example, knockdown of the ten-eleven translocation 3 (TET3) gene of methylcytosine dioxygenase in pyramidal neurons of the DH was found to decrease the activation of pyramidal neurons, thus leading to the impairment of Pavlovian CPP memory reconsolidation (Liu et al., 2018). A putative explanation is that DNA demethylation promotes the binding of transcription factors (Miller et al., 2010; Jarome and Lubin, 2014), which in turn regulate the synthesis of new proteins necessary for memory updating. In another study, using a cocaine operant self-administration (SA) model, Massart et al. (2015) found that incubated cue-induced cocaine-seeking behavior was significantly reduced if the DNA methyltransferase (DNMT) inhibitor (RG108) was delivered intra-NAc on abstinence day 29 and immediately before the extinction test on day 30. This phenomenon could be reversed by the methyl donor S-adenosylmethionine (Massart et al., 2015), indicating that DNMT is a core target for relapse prevention. These results suggest that the signal cascade induced by memory reactivation opened a temporal window during which drug-related memories could be disrupted. In this study, the DNMT inhibitor RG108 was initially delivered one day before CS exposure and again immediately before CS presentation. In contrast, intra-BLA injection of the DNMT inhibitor 5-azacytidine (5-AZA) immediately after CS exposure, but not after a 6-h delay, disrupted cocaine memory reconsolidation (Shi et al., 2015). These two experiments reported similar results in spite of differences in the drug intervention times used, suggesting a complex mechanism underlying reconsolidation that will require more results to further clarify.

Experiments targeting the downstream gene, protein of the CDK5 (cyclin-dependent kinase 5), also found lower expression of the incubation of cocaine craving (Massart et al., 2015). Moreover, in another study, targeting protein kinase CDK5 in the BLA (but not CeA) immediately after memory reactivation, but not after a 6-h delay, abolished a cocaine CPP (Li et al., 2010). These studies suggest that DNA methylation is a core target for preventing relapse, and that protein expression, but not transcription, must be targeted within a specific reconsolidation time window (Nader et al., 2000a; Sorg et al., 2015).

Acetylation and deacetylation of chromatin is another signaling pathway involved in regulating the formation of drug context-associated memories contributing to addiction-like behaviors (Rogge et al., 2013; Bender and Torregrossa, 2020; Campbell et al., 2021). These processes are regulated by two kinds of functionally similar enzymes. Histone acetyltransferases (HATs) facilitate transcription (Korzus et al., 2004; Kouzarides, 2007; Barrett et al., 2011), while histone deacetylases (HDACs) repress transcription (Kumar et al., 2005; Kouzarides, 2007). Both HATs and HDACs are involved in long-term memory formation, and are necessary for Pavlovian cocaine memory consolidation (Malvaez et al., 2011; Taniguchi et al., 2012; Rogge et al., 2013). For example, mice showed significantly improved CPP acquisition after homozygous HDAC3 deletions, due to increased gene expression of c-Fos and nuclear receptor subfamily 4 group A member 2 (Nr4a2) (Rogge et al., 2013). As histone acetylation is required for drug-induced neuroplasticity, later studies have demonstrated that HATs and HDACs are actively involved in drug memory reconsolidation. For example, cue-induced cocaine reinstatement could be enhanced by infusion of trichostatin A (an HDAC inhibitor) into lateral amygdala (LA), and was disrupted by the amnestic agent garcinol (Monsey et al., 2019). These results suggest that changes in histone acetylation are required for memory reconsolidation specifically in the LA, and that targeting HDACs is a possible way to disrupt reconsolidation.

As epigenetic changes affect the initial stage of protein synthesis upstream of transcription, epigenetic manipulation often leads to non-specific consequences. To improve the therapeutic potential of the targeting epigenetic processes, downstream targets and their roles in drug relapse should be identified by future research.

### Autophagy Is a Potent Target for Modifying Drug Memory

Autophagy is an essential pathway for maintaining proteostasis and plays a critical role in neuroplasticity (Liang, 2019). However, direct evidence of autophagy in drug memory consolidation and reconsolidation is rarely reported, although several studies have indicated that autophagy is involved in fear memory consolidation and reconsolidation. For example, one study found that autophagy is recruited in auditory fear memory consolidation by regulating inhibitory neurotransmission via GABA(A)R-associated protein (GABARAP) and its interaction with the GABA(A)R γ2 subunit (Li et al., 2019). Moreover, fear memory reactivation could be prevented by inhibiting synaptic protein degradation (Lee et al., 2008). This effect could be reversed by autophagy-induced synaptic α-amino-3-hydroxy-5-methyl-4-isoxazolepropionic acid receptor (AMPAR) endocytosis (Shehata et al., 2018), suggesting that autophagy may participate in memory reconsolidation via synaptic protein degradation. These reports reveal that the GABAR and AMPAR are potent targets for modulating memory consolidation and reconsolidation. A few studies have also reported that autophagy is required during the degradation of endocytosed GABARs in Caenorhabditis elegans (Rowland et al., 2006) and the degradation of AMPARs in hippocampal neurons (Shehata et al., 2012). As both the GABAR and AMPAR are required for learning and memory (Luo et al., 2015; Roth et al., 2020; Davenport et al., 2021), this presents an ideal opportunity to verify whether autophagy is involved in drug memory consolidation and reconsolidation. However, one pitfall of this approach may be that, as implied in the study (Shehata et al., 2018), autophagy does not directly disrupt reconsolidation, but rather helps to enhance memory destabilization, thus leading to changes in reconsolidation-resistant memories.

### Beta-Adrenergic Signaling Is a Promising Safe Target for Preventing Relapse

Two potential explanations for the limited efficacy of reconsolidation-based pharmacological therapy in preventing relapse are the adverse effects of the amnestic agents administered and the huge surgical trauma involved in targeting specific brain areas. However, these challenges can be addressed by using an amnestic agent with little toxicity that can be delivered in a safe way. Propranolol, a non-specific β-AR blocker, has been reported to be a promising candidate for preventing nicotine, heroin, and cocaine relapse in the clinic (Zhao et al., 2011; Saladin et al., 2013; Xue et al., 2017b; Chen et al., 2021). Furthermore, propranolol has been shown to cross the blood–brain barrier and target β-ARs in the amygdala to modulate drug memory reconsolidation (Otis et al., 2015; Zhu et al., 2018). Propranolol has few side effects and has proved powerful in reducing drug craving. Traditionally, to reduce relapse, the association between drug-paired cues and drug reward is targeted to affect CS-induced reconsolidation. However, the application of this approach may be limited as environmental cues are diverse and the extinguished response to cues may be reinstated with the passage of time (Luo et al., 2015). Nevertheless, the limitations of targeting CS-induced reconsolidation could be addressed by also targeting US-induced reconsolidation (Luo et al., 2015). Propranolol is capable of disrupting US-induced reconsolidation via beta-adrenergic signaling (Xue et al., 2017a,b; Deng et al., 2020), making this signaling pathway a promising target for the prevention of relapse.

### Editing Drug Memory at the Synaptic Level

Synaptic plasticity plays an essential role in neuroadaptations caused by drug addiction and the subsequent maladaptive learning (Kauer and Malenka, 2007). Drug abuse induces changes in synaptic strength, known as synaptic plasticity, to support the formation of associative memories between environment cues and drug reward. Technological advances in experimental methods have allowed precise observation of the process of reconsolidation. For example, one study observed that recognizing training contexts more precisely and more effectively during fear memory retrieval required transiently increased excitation of engram cells (neuroplasticity) (Pignatelli et al., 2019), indicating the dynamic nature of synapses during reconsolidation. Furthermore, in a SA model, researchers found that cocaine memory retrieval promoted the re-maturation of matured silent synapses during the destabilization window (Wright et al., 2020). Blocking silent synapse re-maturation in the NAc during this window gave rise to reduced cocaine-seeking behavior after cue exposure, suggesting that synaptic plasticity is required for memory reconsolidation. Intriguingly, independent of memory retrieval, Young et al. (2016) found that context-induced drug-seeking was disrupted by preventing synaptic actin polymerization in methamphetamine (METH) addiction. In addition, BLA spine dynamics have been shown to contribute to the formation and disruption of METH-associated memory (Young et al., 2020). As the impairment of METH-associated memories was independent of memory reactivation, this approach provides a novel way to edit drug memory outside of the reconsolidation window. These studies reveal the dynamic nature of synaptic plasticity in reconsolidation-dependent, as well as reconsolidation-independent, memory editing.

## Boundary Conditions for Memory Reconsolidation

### Boundaries for Memory Reactivation and Updating

Although drug-paired cues provide effective targets for editing addiction memory, there are several problems that limit reconsolidation-based therapy. Firstly, memory reactivation may be limited due to the requirements for specific retrieval conditions (e.g., context, schedule, or duration of retrieval) or memory features (e.g., age or strength) (Lee, 2009; Lee et al., 2017; Piva et al., 2019). In addition, to promote memory destabilization, a prediction error is usually required (Exton-McGuinness et al., 2015; Sinclair and Barense, 2019). Among people with drug addictions, inter-individual differences may be a boundary condition for the prediction error; this can be attributed to individual drug use histories and incentive value to cues (Kuijer et al., 2020). It would be a significant advance if future research was successful in identifying the biomarkers of drug memory destabilization (Wang et al., 2020). Secondly, CS-induced memory reconsolidation only helps to target specific drug-paired cues (Xue et al., 2012). Although US-induced reconsolidation seems to be a more effective target for preventing relapse (Luo et al., 2015; Dunbar and Taylor, 2017; Xue et al., 2017b), there may be ethical barriers to evoking drug memories using low-dose drug priming in the clinic. Lastly, memory updates may be ineffective in people with drug addictions who also suffer from psychiatric disorders. For instance, in a clinical study, compared with control subjects, patients diagnosed with schizophrenia displayed significant impairment of CS-induced recall of an extinguished memory (Holt et al., 2009).

### Boundaries for Reconsolidation-Based Therapy

Reconsolidation-based therapy has not been appreciably improved by neuroscientific research. One boundary limiting this is that mechanistic studies using rodent models do not combine volitional social factors (Heilig et al., 2016). Recently, Venniro et al. (2018, 2019) and Venniro and Shaham (2020) introduced social context into the classic SA model, demonstrating that operant social interaction could prevent drug addiction, broadening the horizon of relapse prevention. To prevent relapse, methodological innovation is needed in order to provide new ways to understand addiction and to control drug abuse.

## Conclusion

Over the last two decades, reconsolidation theory has progressed from a topic of debate to a basis for clinical therapy. Fundamental research in rodents has revealed the brain regions and molecular processes recruited during reconsolidation. However, boundary conditions limiting progress in memory destabilization and clinical translation remain a challenge for neurobiologists. Meanwhile, reports implying that drug memories can be modified without memory reactivation could provide a promising supplementary approach to reconsolidation-based therapy (Young et al., 2016, 2020). With the development of novel techniques and the accumulation of scientific evidence, we keep an open mind with regard to the potential role of reconsolidation theory in drug relapse prevention.

## Author Contributions

LC developed the manuscript, corrected the style, reviewed and edited the manuscript, and discussed the central ideas of it. HY, YW, XF, SH, and FW developed the manuscript and discussed the central ideas of it. ZH and QL designed the graph and reviewed and edited the manuscript. JY developed the manuscript, proposed the central idea of it, reviewed and edited the manuscript, and acquired funding. All authors contributed to the article and approved the submitted version.

## Conflict of Interest

The authors declare that the research was conducted in the absence of any commercial or financial relationships that could be construed as a potential conflict of interest.

## Publisher’s Note

All claims expressed in this article are solely those of the authors and do not necessarily represent those of their affiliated organizations, or those of the publisher, the editors and the reviewers. Any product that may be evaluated in this article, or claim that may be made by its manufacturer, is not guaranteed or endorsed by the publisher.
