# Corrigendum: The Mechanisms and Boundary Conditions of Drug Memory Reconsolidation

**DOI:** 10.3389/fnins.2021.758136

**Published:** 2021-09-07

**Authors:** Liangpei Chen, He Yan, Yufang Wang, Ziping He, Qihao Leng, Shihao Huang, Feilong Wu, Xiangyang Feng, Jie Yan

**Affiliations:** ^1^Department of Forensic Science, School of Basic Medical Science, Central South University, Changsha, China; ^2^Xiangya School of Medicine, Central South University, Changsha, China; ^3^Key Laboratory of Molecular Epidemiology of Hunan Province, School of Medicine, Hunan Normal University, Changsha, China; ^4^Department of Forensic Science, School of Basic Medical Science, Xinjiang Medical University, Urumqi, China

**Keywords:** drug memory, addiction, reconsolidation, limbic–corticostriatal system, boundary condition

Contributions of the first author were incorrectly labeled as Liangpei Chen^1†^. The correct label is Liangpei Chen^1^. Liangpei Chen is the only first author. The symbol “†” (representing these authors contributed equally) of the author bar in the article is redundant.

The authors apologize for this error and state that this does not change the scientific conclusions of the article in any way. The original article has been updated.

## Publisher's Note

All claims expressed in this article are solely those of the authors and do not necessarily represent those of their affiliated organizations, or those of the publisher, the editors and the reviewers. Any product that may be evaluated in this article, or claim that may be made by its manufacturer, is not guaranteed or endorsed by the publisher.

